# Association between exposure to terpene compounds and risk of metabolic syndrome: exploring the potential mediating role of inflammatory response

**DOI:** 10.3389/fendo.2025.1551784

**Published:** 2025-05-06

**Authors:** Jiyu Nie, Zhizhuo Huang, Lin Wen, Haiying Li, Qianqian Xie, Houchun Wang, Zhengtian Lai, Chuhang Lin, Chunxia Jing

**Affiliations:** ^1^ Department of Public Health and Preventive Medicine, School of Medicine, Jinan University, Guangzhou, Guangdong, China; ^2^ Guangdong Key Laboratory of Environmental Exposure and Health, Jinan University, Guangzhou, Guangdong, China; ^3^ Department of Medical Records, Guangdong Provincial People’s Hospital (Guangdong Academy of Medical Sciences), Southern Medical University, Guangzhou, China; ^4^ Guangzhou University of Chinese Medicine, The Second Clinical Medical College, Guangzhou, Guangdong, China

**Keywords:** terpene, metabolic syndrome (MetS), mixed exposure, BKMR, QGC

## Abstract

**Background:**

Terpenes are potentially harmful substances that are associated with endocrine disruption due to their ability to produce oxidizers, aldehydes, and secondary aerosol particles. However, the exact association between terpenoids and metabolic syndrome remains unclear.

**Objective:**

This study aims to examine the relationship between individual and mixed exposure to terpene compounds and the risk of developing metabolic syndrome.

**Methods:**

We utilized data from the NHANES 2013-2014 cycle, including 1,135 participants. Multiple regression models, Bayesian kernel regression (BKMR), and quantile g calculation (QGC) were employed to assess the association between individual and mixed terpene exposure and metabolic syndrome. Additionally, a mediation analysis was performed to explore potential biological pathways mediated by inflammation, using the Advanced Cancer Inflammation Index as a metric.

**Results:**

The regression analysis indicated a positive association between exposure to limonene and metabolic syndrome (OR (95%):1.74(1.17, 2.57), p=0.005). The BKMR regression and the QGC model showed a positive association between exposure to mixed terpenes and the increased risk of metabolic syndrome (p=0.001). Subgroup analyses within the BKMR revealed significant positive trends among males, individuals under 60, and the overweight groups. Furthermore, exposure to mixed terpenes exhibited positive trends with lower HDL levels(p<0.000). The Advanced Cancer Inflammation Index was identified as a potential mediator of the positive correlation between α-pinene, β-pinene, and metabolic syndrome.

**Conclusions:**

This study suggests that exposure to both individual and mixed terpenes may increase risk of developing metabolic syndrome. However, further longitudinal studies are imperative to establish causality between terpene compounds and the risk of metabolic syndrome.

## 1 Introduction

Metabolic syndrome is a cluster of symptoms, including insulin resistance, abdominal obesity, dyslipidemia, and elevated blood pressure (1). Recent data from the American Heart Association’s updated NCEP III definition showed that approximately 35 percent of the American population exhibit symptoms of metabolic syndrome, and this number continues to rise (2). Several studies have consistently demonstrated that metabolic syndrome amplifies the probability of developing cardiovascular disease by twofold and greatly augments the risk of developing type 2 diabetes by fivefold (3, 4). In addition to traditional risk factors like unhealthy diet and sedentary lifestyles, exposure to environmental pollutants has also been shown to affect the development of metabolic syndrome (5–7).

Terpenes, particularly monoterpenes (MTs) like as α-pinene, β-pinene, and limonene, are commonly found as trace gases in the troposphere (8). These compounds are prevalent in household cleaning products, air fresheners, and sanitizing products, making them significant sources of indoor air pollutants and personal exposures (9). Furthermore, terpenes are often added to e-cigarette liquids as flavoring agents (10). A survey of U.S. households showed that a vast majority (99.1%) of Americans are exposed to terpene-containing scented products on a weekly basis (11). A study by Steinemann (12) found that 156 scented consumer products emit more than 37 volatile organic compounds (VOCs), with major compounds including limonene, α-pinene, β-pinene, and other terpenes. Surprisingly, exposure to these scented products has resulted in adverse health effects for more than one-third of Americans. However, the specific compounds produced by these products are not disclosed to the public (13). Because monoterpenes have low solubility in water but high solubility in blood and lipophilic tissues (14), exposure to compounds like α-pinene can result in elevated blood concentrations. Therefore, serum levels of terpenes are commonly used as indicators for assessing environmental terpene exposure (15).

When individuals are exposed to different aldehydes, which are generated through the secondary reaction of terpene compounds with airborne ozone, it can activate an inflammatory response that escalates the likelihood of developing metabolic syndrome (16). Previous studies have consistently shown that exposure to aldehyde is associated with a range of negative health consequences, such as elevated blood pressure, increased risk of cardiovascular events, adult obesity, and metabolic syndrome (17). In particular, Yan et al. found that high serum levels of aldehyde, specifically isovaleric aldehyde, are linked to an elevated risk of metabolic syndrome (18). The second mechanism suggests that exposure to indoor terpenes leads to oxidation through the presence of airborne ozone and hydroxyl radicals. This oxidative process generates a range of organic pollutants in the environment, which then accumulate and contribute to the formation of secondary aerosolized particulate matter(SOA) (19). Previous studies have shown that secondary organic aerosols are an important component of fine particulate matter, and that terpene exposure results in significantly elevated levels of fine particulate matter, the inhalation of which may lead to elevated metabolic syndrome (20). Moreover, previous studies have shown that apolipoprotein B (Apo-B) partially mediates the relationship between SOA and the risk of metabolic syndrome (21). Research has also indicated that SOA triggers the production of reactive oxygen species (ROS) in replacement lung fluid (SLF), causing oxidative stress and inflammation *in vivo (* 22). It has also been observed that this inflammation is linked to an increased risk of developing diabetes and metabolic syndrome (23, 24).

It is reasonable to assume that using cleaning products containing terpenes can lead to significant exposure to these compounds, which produce aldehydes and SOA that disrupt the body’s natural metabolism and contribute to the development of metabolic syndrome. To comprehensively evaluate the impact of terpenoids on metabolic syndrome, this study employed three statistical models: linear regression, Bayesian kernel-mechanism regression, and quantitative g-calculation models to analyze the individual and mixed effects of exposure to terpene. Furthermore, we chose to investigate the mediating role of inflammatory effects (as indicated by the Advanced Cancer Inflammation Index (ALI)) in the relationship between terpene and the metabolic syndrome, which can provide abundant evidence and stimulate new ideas for further exploration of this pathway. The ALI is an innovative marker that combines measures of inflammation and nutrition (25). Initially developed as an independent prognostic indicator in advanced cancer, the ALI is now widely used as a composite index of nutrition and inflammation in the general U.S. population. The data used in this study come from the National Health and Nutrition Examination Survey (NHANES) 2013-2014, ensuring a reliable and robust foundation for analysis.

## 2 Method

### 2.1 Study population

The National Health and Nutrition Examination Survey (NHANES) is a comprehensive research program designed to evaluate individuals’ health and nutritional status across different age groups in the United States. The NHANES program employs a sophisticated multistage probability sampling strategy that selectively samples non-hospitalized civilians, enhancing the representativeness of the obtained data. The NHANES protocol was approved by the NCHS Research Ethics Review Board, and each participant provided written consent prior to participating in the study, ensuring the highest standards of scientific integrity and safeguarding the rights and welfare of the research participants.

**Figure 1:**
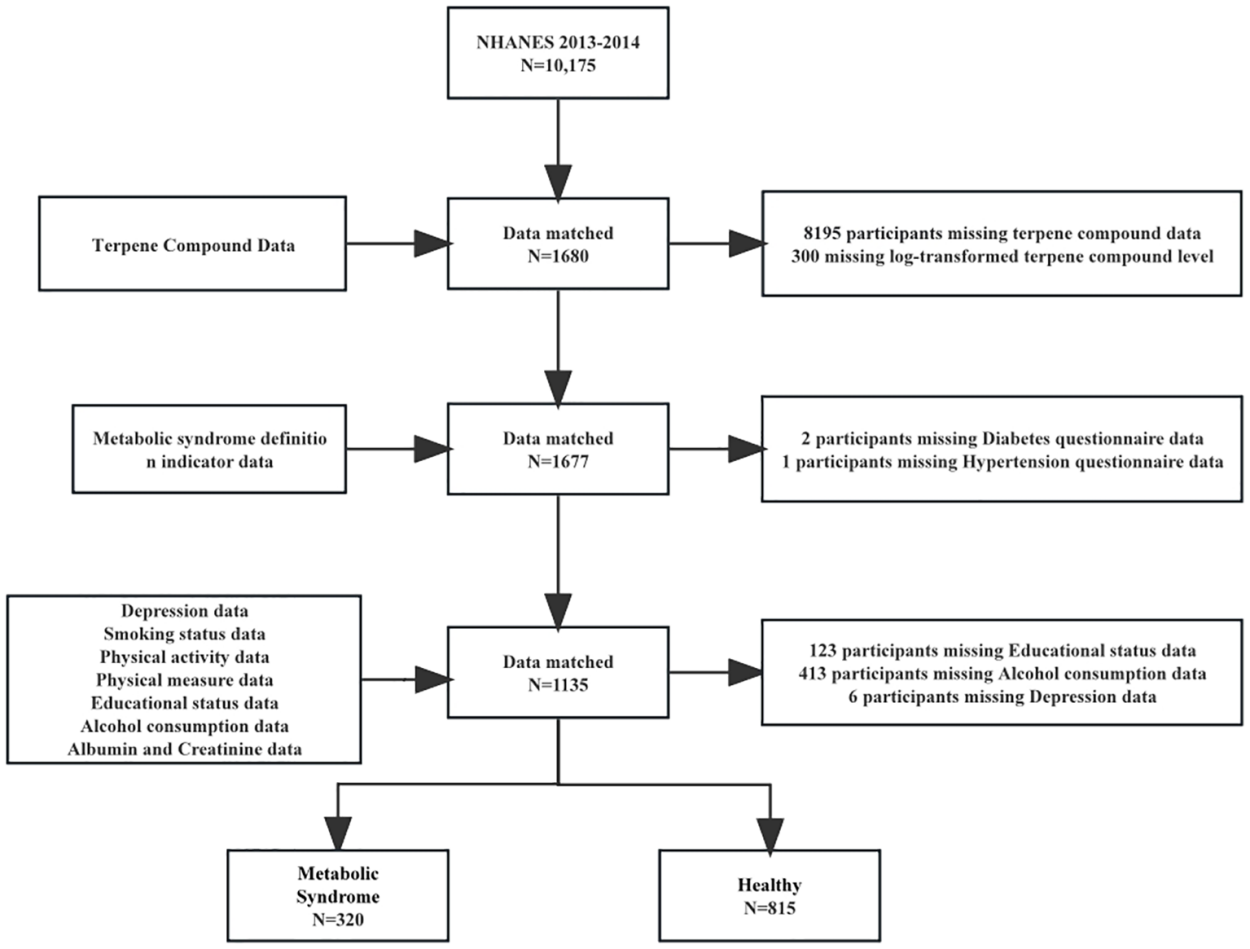
Flowchart for the selection of eligible participants.

In this study, we used cross-sectional data from the NHANES 2013-2014, and 10,175 participants were screened. We merged the databases based on the unique identity of the survey subjects. After merging the databases, we excluded 9,662 who had missing data in alcohol consumption data, diabetes questionnaire data, terpene compound data and educational status data. Finally, 1,136 subjects were included in the study (**Figure 1**). We found that subjects included in the study had a lower body mass index (27.8 versus 29.1 kg/m²), a higher level of education (45.2% versus 31.8%), and a lower prevalence of hypertension (28.1% versus 34.9%) (**Supplementary Table S8**). These differences suggest that the excluded subjects may represent a less healthy population, potentially underestimating the true association between terpene exposure and Mets.

### 2.2 Measurement of serum terpenes

Serum specimens were collected, stored, and shipped to the division of laboratory sciences, National Center for Environmental Health, Centers for Disease Control and Prevention, Atlanta GA, for analysis. The detection of terpenes was based on headspace solid-phase microextraction-gas chromatography-tandem mass spectrometry (HS-SPME-GC-MS/MS) (15). This method required 0.50 mL of serum spiked with 40 µL of isotopically labeled internal standard before being hermetically sealed in a 10-mL SPME vial with a Teflon-lined silicone septum. After automated headspace sampling, the SPME fiber was injected into the GC inlet to undergo separation using a DB-624 column. The eluates were ionized at 70eV in the electron source of the mass spectrometer. The tandem mass spectrometer ran in multiple reaction monitoring (MRM) mode, where the precursor ions fragmented with nitrogen gas in the collision cell at a defined electron voltage. The resultant product ions registered an interpretable signal via the electron multiplier. We compared the relative response ratio (native to internal standard signal) with a calibration curve of known standards to quantify the concentration of the individual terpene in the matrix. All manufacturer-recommended quality control procedures were followed during the serum terpene assay. Reported results for all assays met the laboratory sciences department’s QA/QC performance standards for accuracy and precision, similar to the Westgard Rules.

### 2.3 Definition of metabolic syndrome

Mets are defined according to the criteria in the Executive Summary of the National Cholesterol Education Program (NCEP) Expert Panel’s Third Report on Detection, Evaluation, and Treatment of High Blood Cholesterol in Adults (Adult Treatment Panel III) (26). Participants fulfilling three or more of the following criteria were classified as having Mets: 1) Hypertriglyceridemia (TG): ≥ 1.69 mmol/L (150 mg/dL); 2) Low HDL: < 1.03 mmol/L (40 mg/dL) for men and < 1.29 mmol/L (50 mg/dL) for women; 3) Hyperglycemia: fasting plasma glucose (FPG) ≥ 6.1 mmol/L, or HbA1c level ≥ 6.5%, or receiving glucose-lowering therapy, or using insulin, or diagnosed with diabetes mellitus; 4) Abdominal obesity: waist circumference > 102 cm for men and > 88 cm for women; 5) Hypertension: systolic blood pressure (SBP) ≥ 130 mmHg or diastolic blood pressure (DBP) ≥ 85 mmHg, or receiving antihypertensive therapy, or diagnosed with hypertension by a physician.

### 2.4 Definition of advanced lung cancer inflammation index

The calculation of the ALI score is based on three factors: body mass index (BMI), serum albumin (Alb), and neutrophil-lymphocyte ratio (NLR), which are all markers of systemic inflammation (27, 28). The ALI score, calculated as ALI = BMI × Alb/NLR, integrates markers of systemic inflammation and nutritional status. A lower ALI score reflects higher systemic inflammation due to elevated NLR or reduced BMI/Alb levels, while a higher ALI score indicates lower inflammation risk. This metric has been validated as a proxy for inflammation in chronic diseases, initially in advanced cancer populations (25). Since then, ALI has been applied to other chronic conditions, including predicting coronary artery disease (29), hypertension (30), heart failure in the elderly (31), all of which are closely associated with metabolic syndrome (32). Although ALI provides a composite measure of inflammation and nutrition, its generalizability to non-cancer populations requires further validation, particularly in cohorts with diverse metabolic profiles.

### 2.5 Covariates

Sixteen covariates were included based on their established association with MetS, including demographics (age, sex, race), lifestyle factors (smoking, alcohol use), metabolic parameters (BMI, waist circumference, blood glucose), and comorbidities (diabetes, hypertension). This selection aligns with consensus criteria from Grundy et al. (33) and Alberti et al. (34).

Smoking status was assessed through a self-report questionnaire and classified into three categories: never smoked, ever smoked and current smoker. Participants who answered “No” to the question “Have you smoked at least 100 cigarettes in your lifetime?” were considered as never smoked. Those who answered “Yes” to the same question but “Not at all” to the question “Do you currently smoke cigarettes?” were categorized as ever smoked. Participants who answered “Yes” to the question “Have you smoked at least 100 cigarettes in your lifetime?” and “Every day” or “Some days” to the question “Do you currently smoke cigarettes?” were classified as current smokers. Hypertension was defined based on participants’ self-report by answering “Yes” to the question “Have you ever been told you have hypertension?” or current use of antihypertensive medication by answering “Yes” to the question “Are you currently taking prescription medication for hypertension?”. Alcohol consumption was determined by participants reporting if they have consumed at least 12 drinks in the past year by answering “Yes”.

### 2.6 Statistical analysis

Continuous variables are presented as means and standard deviations (Mean ± SD), while categorical variables are displayed as cases (n) and percentages (%). We log-transformed the serum terpenes content to ensure normal distribution. Multiple linear regression was performed to analyze the relationship between serum terpenes and Mets. Each serum terpenes were considered as a separate predictor to assess its association with Mets in a representative U.S. population We fit two models to evaluate model stability. Model 1 did not adjust for any covariates, while Model 2 included all covariates. Considering the limitations of linear regression for high-dimensional data and nonlinear exposure-outcome relationships, we also utilized BKMR and QGC methods to estimate the mixed effects of serum terpenes exposure on Mets.

BKMR models generate posterior inclusion probabilities (PIPs) that range from 0 to 1, indicating the relative contribution of each monoterpene to Mets (35). This approach provides a flexible method to model the multifaceted effects of mixed substances, allowing for interactions between substances and capturing non-linear relationships between exposure and outcome using exposure-response curves (36). A variable selection method was performed using a Markov Chain Monte Carlo (MCMC) algorithm with 50,000 iterations. We also explore the interaction between three terpenes by fixing them at the 50th percentile and plotting the bivariate exposure-response functions for each terpene at the 25th, 50th, and 75th percentiles (**Supplementary Figure S3**).

The QGC model was employed in this study. We estimated the weights for both ψ (represents a parameter) and the degree of violation of the assumption of directional homogeneity (37). The findings of this study illustrate that this approach enables robust inferences to be made regarding the effects of exposure to the entire mixture and the contributions of individual components, especially in cases where directional homogeneity cannot be assumed (38). Furthermore, we conducted a mediation analysis to examine the impact of advanced cancer inflammation index (ALI) on the relationship between limonene, α-pinene, β-pinene, and metabolic syndrome.

Given the exploratory nature of this study, we did not apply multiple testing corrections (e.g., Bonferroni, FDR) to maintain sensitivity for detecting potential associations. However, results should be interpreted with caution, as unadjusted analyses may increase the risk of Type I errors.

In this study, a significance level of 0.05 was employed to establish the threshold for statistical significance. All statistical analyses were performed using STATA (Version 15.1) and R software (Version 4.2.2).

## 3 Results

### 3.1 Demographic characteristics

The study includes 1,135 participants, out of which 320 individuals (28.1% of the total sample) were diagnosed with Mets. The results showed a statistically significant difference in age between the healthy and Mets groups (p<0.001). Additionally, the Mets group exhibited a higher proportion of older individuals compared to the healthy group, with 40.3% versus 26.0%. There were also significant differences between the healthy and Mets groups in marital status, education level, smoking and drinking habits, physical exercise, and BMI. Compared to the healthy group, the Mets group had a higher proportion of individuals with hypertension (70.9% vs. 23.2%), diabetes (40.3% vs. 2.8%), cardiovascular disease (19.4% vs. 6.1%), hyperuricemia (35.3% vs. 14.0%), and NAFLD (85.3% vs. 45.4%) (**Table 1**).

**Table 1:** Demographic and individual characteristics of the study population(N=1,135).

	Metabolic Syndrome (N=320)	Healthy (N=815)	P-value
Age group (n(%))			
20~60 years	191 (59.7%)	603 (74.0%)	<0.001***
Over 60 years	129 (40.3%)	212 (26.0%)	
Sex (n(%))			
Male	154 (48.1%)	440 (54.0%)	0.086
Female	166 (51.9%)	375 (46.0%)	
Race (n(%))			
Mexican American	46 (14.4%)	87 (10.7%)	0.099
Other Hispanic	33 (10.3%)	61 (7.5%)	
Non-Hispanic White	161 (50.3%)	424 (52.0%)	
Non-Hispanic Black	53 (16.6%)	146 (17.9%)	
Other Race - Including Multi-Racial	27 (8.4%)	97 (11.9%)	
Marital status (n(%))			
Married/Living with partner	203 (63.4%)	471 (57.8%)	<0.001***
Never married	41 (12.8%)	187 (22.9%)	
Widowed/Divorced/Separated	76 (23.8%)	157 (19.3%)	
Education level (n(%))			
Under high school	67 (20.9%)	125 (15.3%)	0.003**
High school or equivalent	87 (27.2%)	179 (22.0%)	
Above high school	166 (51.9%)	511 (62.7%)	
Ratio of Family Income to Poverty (n(%))			
Poverty	68 (21.3%)	162 (19.9%)	0.663
Above Poverty	252 (78.8%)	653 (80.1%)	
Smoking (n(%))			
Never	153 (47.8%)	420 (51.5%)	0.036*
Ever	105 (32.8%)	207 (25.4%)	
Current	62 (19.4%)	188 (23.1%)	
Drinking (n(%))			
Less than 12 alcoholic beverages/1 year	253 (79.1%)	690 (84.7%)	0.029*
12 alcoholic beverages/1 year or more	67 (20.9%)	125 (15.3%)	
Exercise (n(%))			
Not exercising regularly	187 (58.4%)	367 (45.0%)	<0.001***
Exercise regularly	133 (41.6%)	448 (55.0%)	
Hypertension (n(%))			
Yes	227 (70.9%)	189 (23.2%)	<0.001***
No	93 (29.1%)	626 (76.8%)	
Diabetes (n(%))			
Yes	129 (40.3%)	23 (2.8%)	<0.001***
No	191 (59.7%)	792 (97.2%)	
Depression (n(%))			
Yes	37 (11.6%)	65 (8.0%)	0.074
No	283 (88.4%)	750 (92.0%)	
Cardiovascular Disease (n(%))			
Yes	62 (19.4%)	50 (6.1%)	<0.001***
No	258 (80.6%)	765 (93.9%)	
Hyperuricemia (n(%))			
Yes	113 (35.3%)	114 (14.0%)	<0.001***
No	207 (64.7%)	701 (86.0%)	
Nonalcoholic Fatty Liver Disease (n(%))			
Yes	273 (85.3%)	370 (45.4%)	<0.001***
No	47 (14.7%)	445 (54.6%)	
Body mass index			
Mean (SD)	2.57 (0.609)	1.90 (0.805)	<0.001***
Median [Min, Max]	3.00 [1.00, 3.00]	2.00 [1.00, 3.00]	
Urine creatinine (n(%))			
Mean (SD)	4.54 (0.696)	4.47 (0.768)	0.131
Median [Min, Max]	4.62 [2.20, 5.91]	4.58 [1.61, 6.30]	
α_Pinene (ng/mL)			
Tertile1[n(%)]	67 (20.9%)	217 (26.6%)	0.022*
Tertile2[n(%)]	74 (23.1%)	218 (26.7%)	
Tertile3[n(%)]	85 (26.6%)	200 (24.5%)	
Tertile4[n(%)]	94 (29.4%)	180 (22.1%)	
β_Pinene (ng/mL)			
Tertile1[n(%)]	66 (20.6%)	228 (28.0%)	0.020*
Tertile2[n(%)]	81 (25.3%)	204 (25.0%)	
Tertile3[n(%)]	80 (25.0%)	205 (25.2%)	
Tertile4[n(%)]	93 (29.1%)	178 (21.8%)	
Limonene (ng/mL)			
Tertile1[n(%)]	70 (21.9%)	216 (26.5%)	0.052
Tertile2[n(%)]	73 (22.8%)	214 (26.3%)	
Tertile3[n(%)]	82 (25.6%)	200 (24.5%)	
Tertile4[n(%)]	95 (29.7%)	185 (22.7%)	

*, *P* < 0.05; **, *P* < 0.01; ***, *P* < 0.001. *P*-t, *p*-value for trend. Bold values indicate statistically significant differences between the metabolic syndrome group and the healthy control group (p<0.05).

**Supplementary Table S1** presents the percent detectable, geometric mean, weighted mean, and percentage of detectable terpenes with detection levels greater than or equal to the LOD for the terpenes. Additionally, a correlation heat map of terpenes is shown in **Supplementary Figure S1**, and the results revealed a strong positive correlation (r = 0.75, P < 0.001) between α-pinene and β-pinene, indicating a high degree of similarity between these two compounds. In contrast, the correlation among the other substances was weak (r < 0.5), although their relationship was statistically significant (P < 0.001). To address the issue of covariance, we categorized α-pinene and β-pinene as a single group within the BKMR model (**Supplementary Figure S1**).

### 3.2 Associate between terpenes and Mets by multiple regression

**Figure 2:**
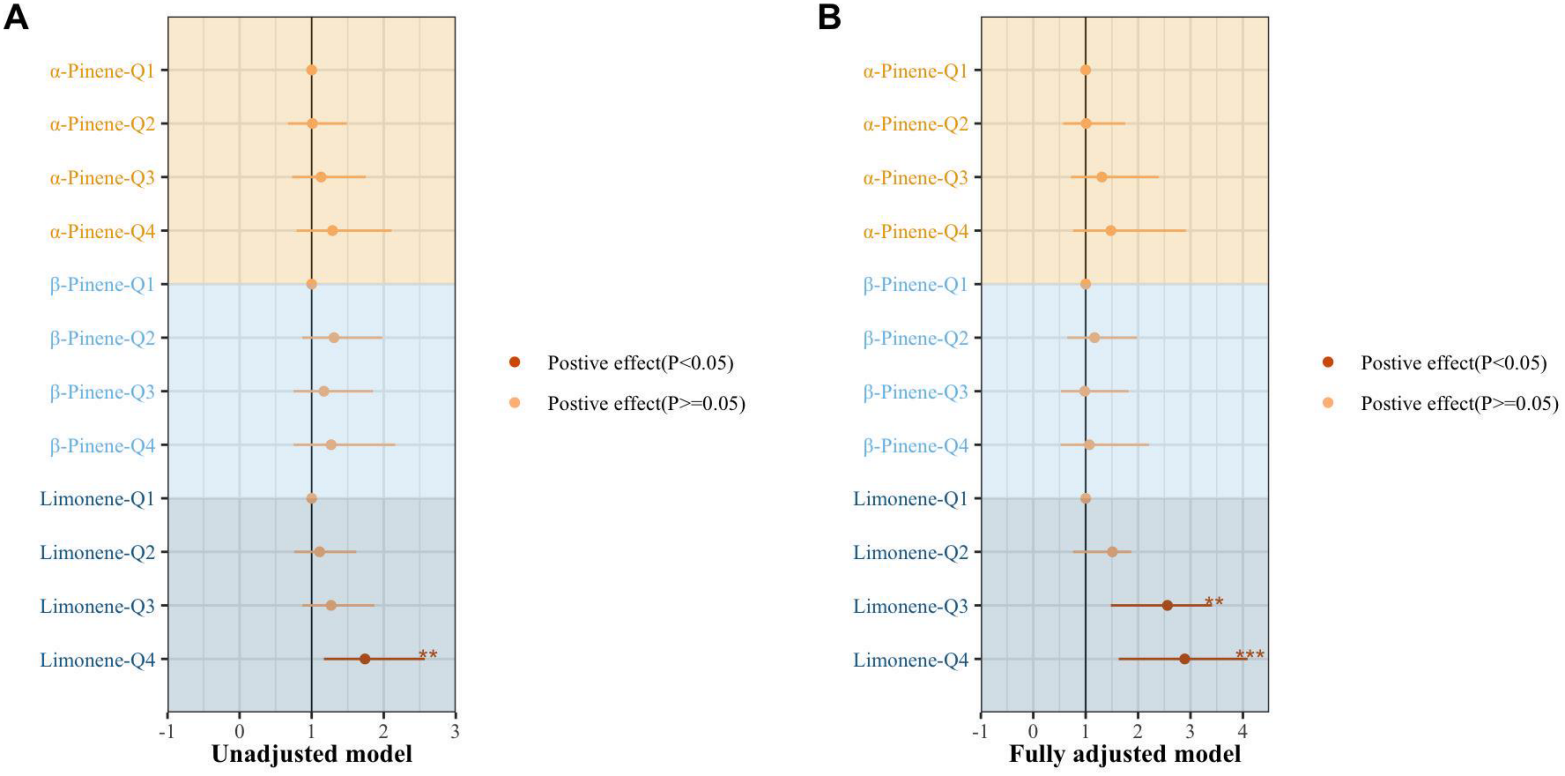
The association of individual terpene with the metabolic syndrome by multiple regression. **(A)** un-adjusted model. **(B)** adjusting for age sex, race, marital status, BMI index, smoking, drinking, exercise, education level, marital status, ratio of family income to poverty, urine creatinine, hypertension, diabetes, depression, cardiovascular disease, nonalcoholic fatty liver disease, and hyperuricemia· *, P < 0.05; **, P < 0.01; ***P < 0.001.

**Figure 2** presents the results of the multiple regression examining the association between individual terpenes and Mets. The analysis revealed that individuals in the fourth quartile of limonene had a 1.74-fold increased risk of developing Mets compared to those in the lowest quartile (95% CI: 1.17-2.57, p=0.005). After adjusting for covariates, the risk of Mets significantly increased in the third and fourth quartiles of limonene, with OR of 2.56 (95% CI: 1.48-3.41, p=0.001) and 2.98 (95% CI: 1.63-4.09, p=0.000), respectively.

### 3.3 Associate between terpenes and Mets using the BKMR model

**Figure 3:**
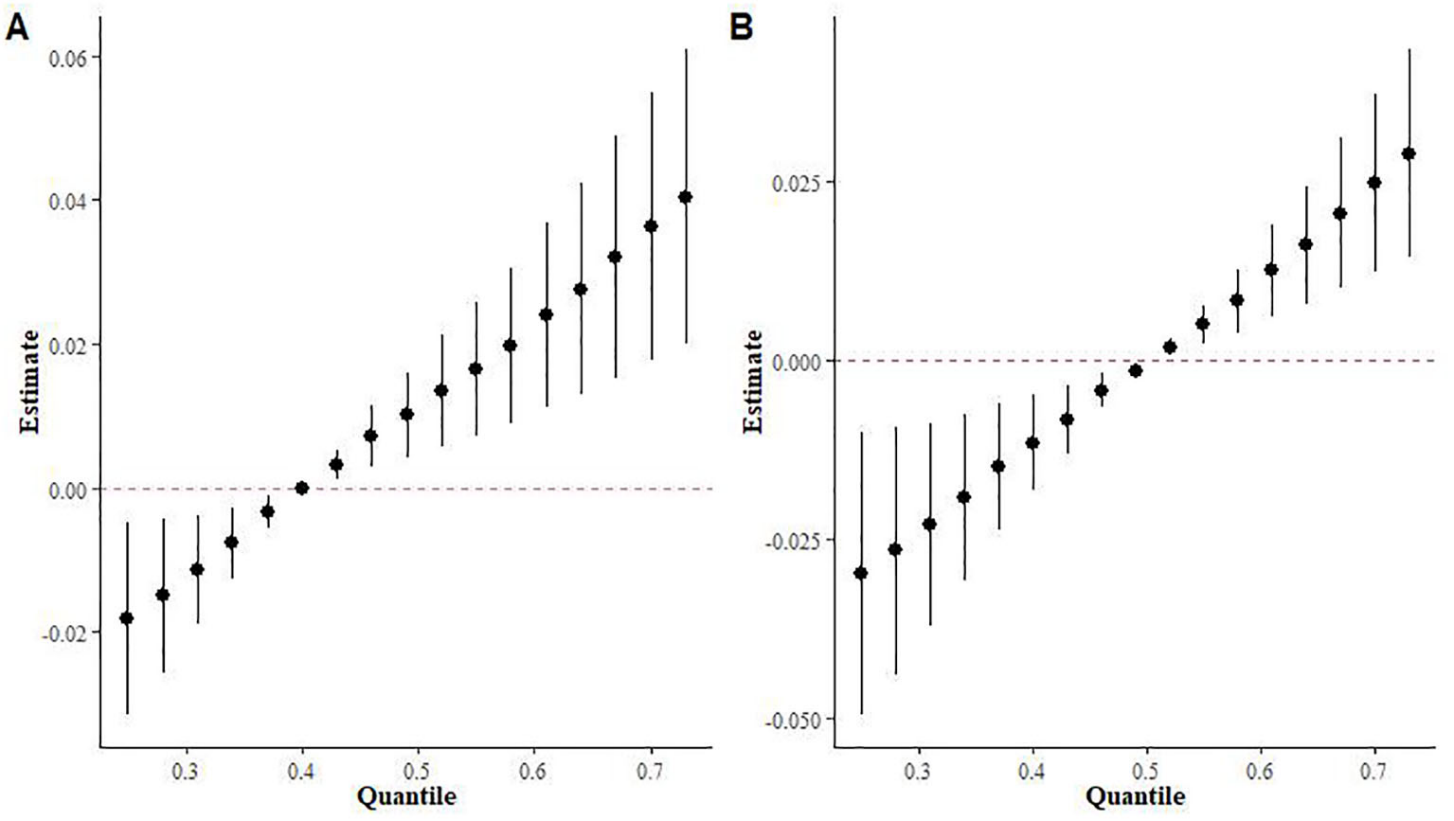
Effects of overall population mixed terpene exposure on metabolic syndrome in the BKMR model. **(A)** Fixed at the 40th percentile. **(B)** Fixed at the 50th percentile.

The BKMR model showed a significant positive correlation between exposure to the three monoterpenes simultaneously and Mets when terpene exposure was set at the 40th and 50th percentiles (**Figure 3**).

**Figure 4:**
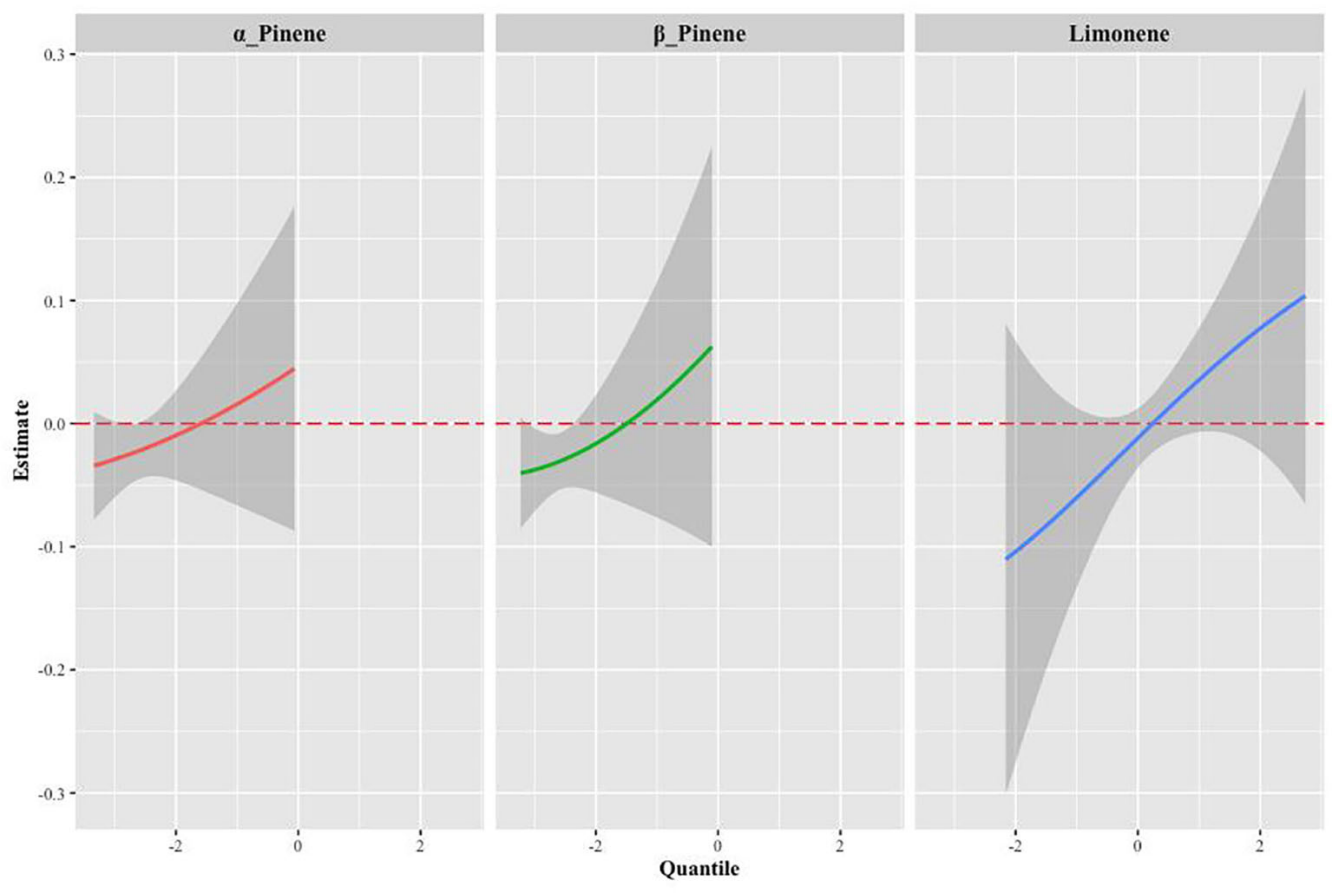
The univariate exposure-response function curves for a single terpene associated with MS, when the other two monoterpenes were fixed at their median values (95% CrI).

**Supplementary Table S2** provides a summary of groupPIP and condPIP for each metal. The second group had the highest groupPIP (PIP=0.955), with limonene showing the most significant contribution (condPIP=1.000), suggesting that it may play a crucial role in the association with Mets. **Figure 4** depicts the univariate exposure-response curves of three terpene derivatives (α-Pinene, β-Pinene, and limonene), demonstrating a distinct positive dose-response relationship.

The results of the BKMR interaction modeling indicate that the slope of the bivariate reaction function for any given terpenes remain constant across the different quantiles of other terpenes, suggesting no potential interaction between the various terpenes (**Supplementary Figure S2**). In BKMR’s univariate effect plot exposure to limonene exhibited a statistically significant positive association with Mets when we controlled other terpene exposures at 0.25,0.5 and 0.75content (**Supplementary Figure S3**).

**Figure 5:**
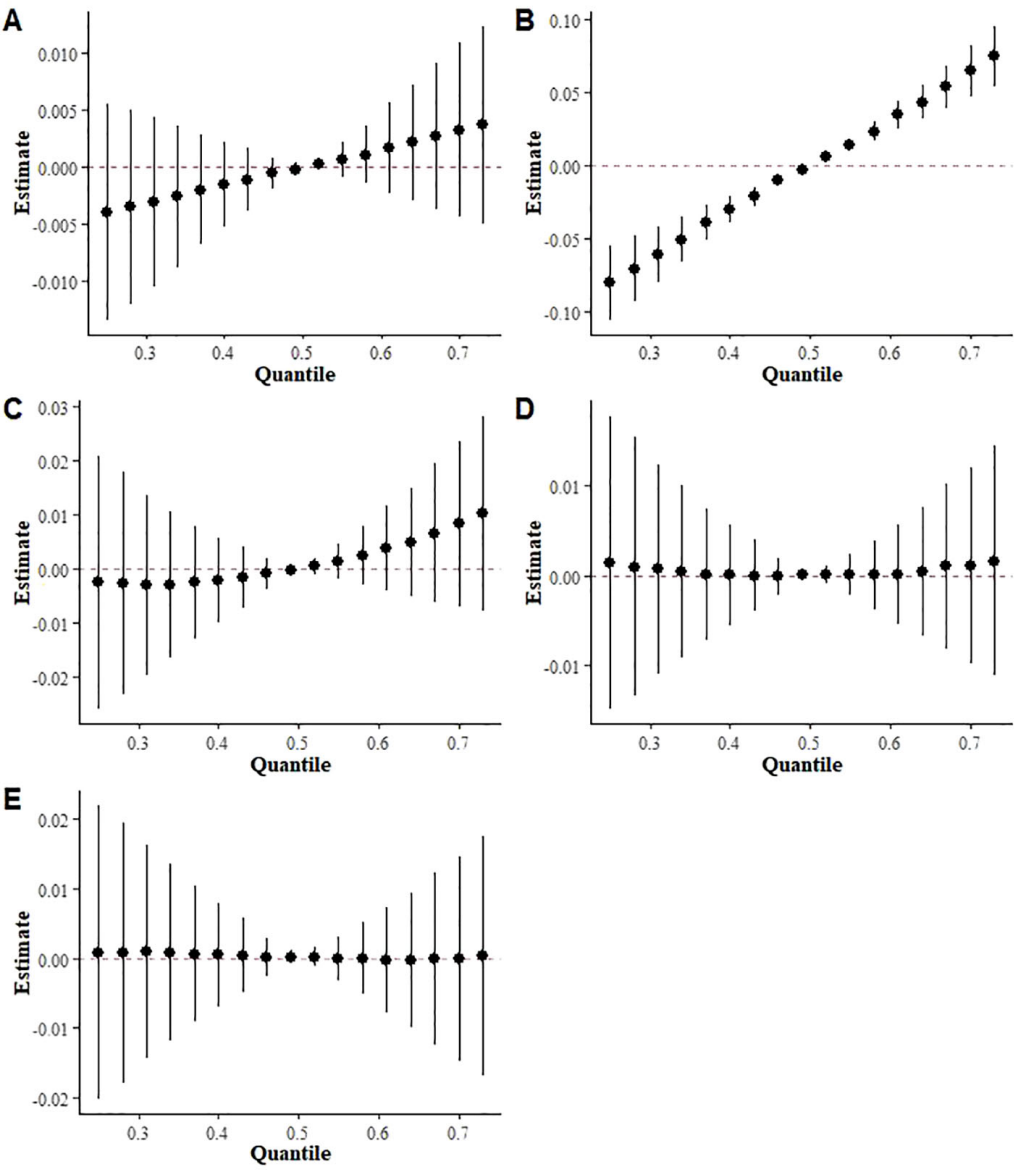
BKMR results of exposure to a mixture of three terpene derivatives on five indices included in the metabolic syndrome (**(A)** High triglycerides, **(B)** Low HDL, **(C)** Abdominal obesity, **(D)** Hypertension **(E)** Diabetes mellitus).

To further investigate the relationship between mixed exposure to the three terpene derivatives and the five metabolic syndrome metrics (high triglycerides, low HDL, abdominal obesity, hypertension, and diabetes mellitus), the BKMR model was used. The analysis revealed a significant positive correlation between mixed terpene exposure and Low HDL (OR (95%):2.51(1.66, 3.80), p<0.000), while the correlation with high triglycerides and high abdominal fat was relatively weak, as shown in **Figure 5**.

### 3.4 Stratified analysis by gender, age, and BMI

**Figure 6:**
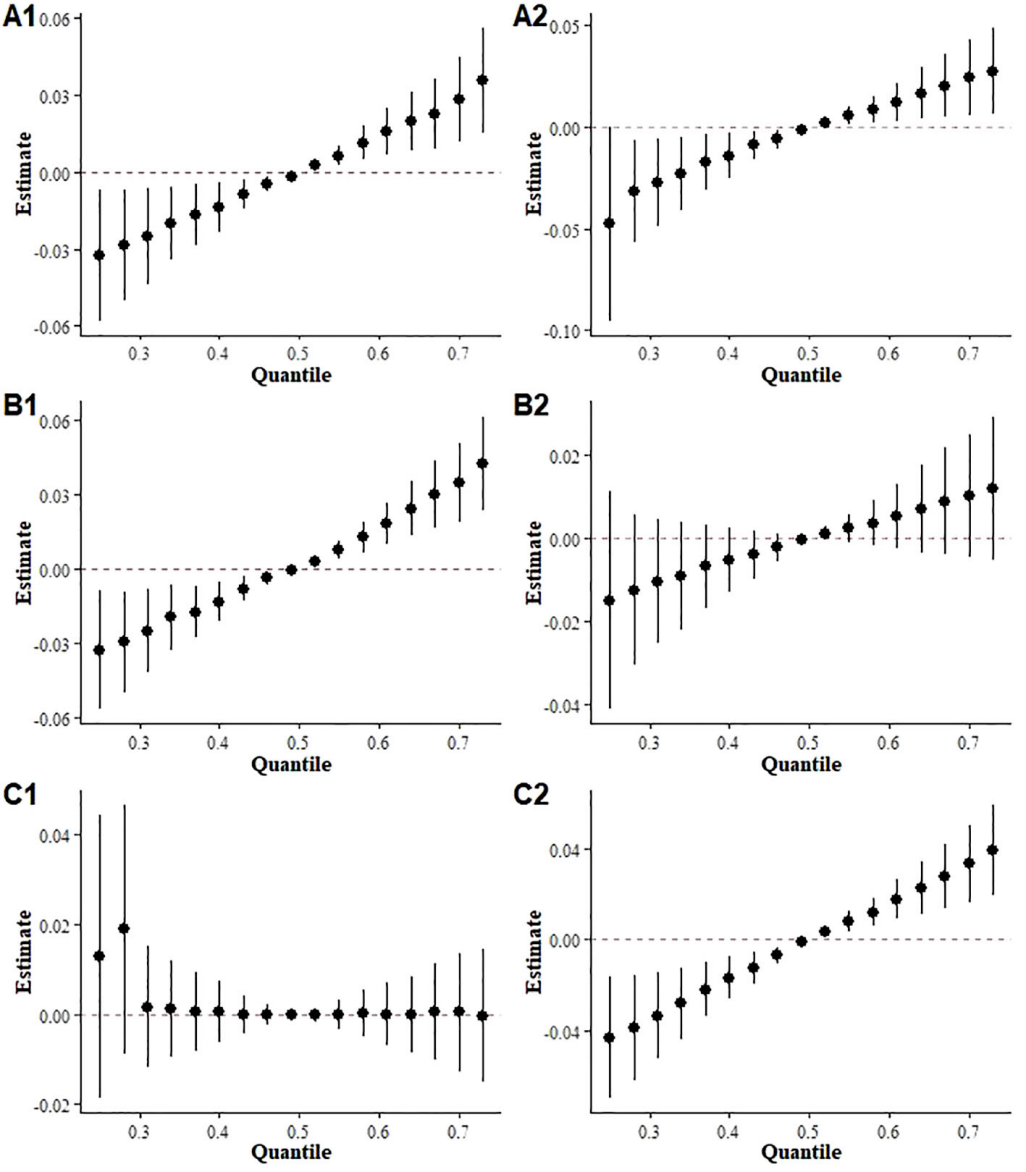
Overall effects of mixed terpene exposure on the metabolic syndrome stratified by subgroup. **(A)** sex (A1: Male, A2: Female); **(B)** age (B1: under 60 years, B2: over 60 years); **(C)** BMI (C1: under 25 kg/m2, C2 over 25 kg/m2).

**Supplementary Tables S3**-**S5** show the results of multiple linear regressions stratified by sex, age, and BMI, suggesting that exposure to mixed terpenes may pose a higher risk of Mets in males, individuals under 60 years of age, and overweight groups. The stratified analysis of the BKMR model also showed a significant positive association between terpene exposure and Mets in both sexes, under 60 and overweight groups (**Figure 6**).

### 3.5 Associate between terpenes and mets using QGC model

**Figure 7:**
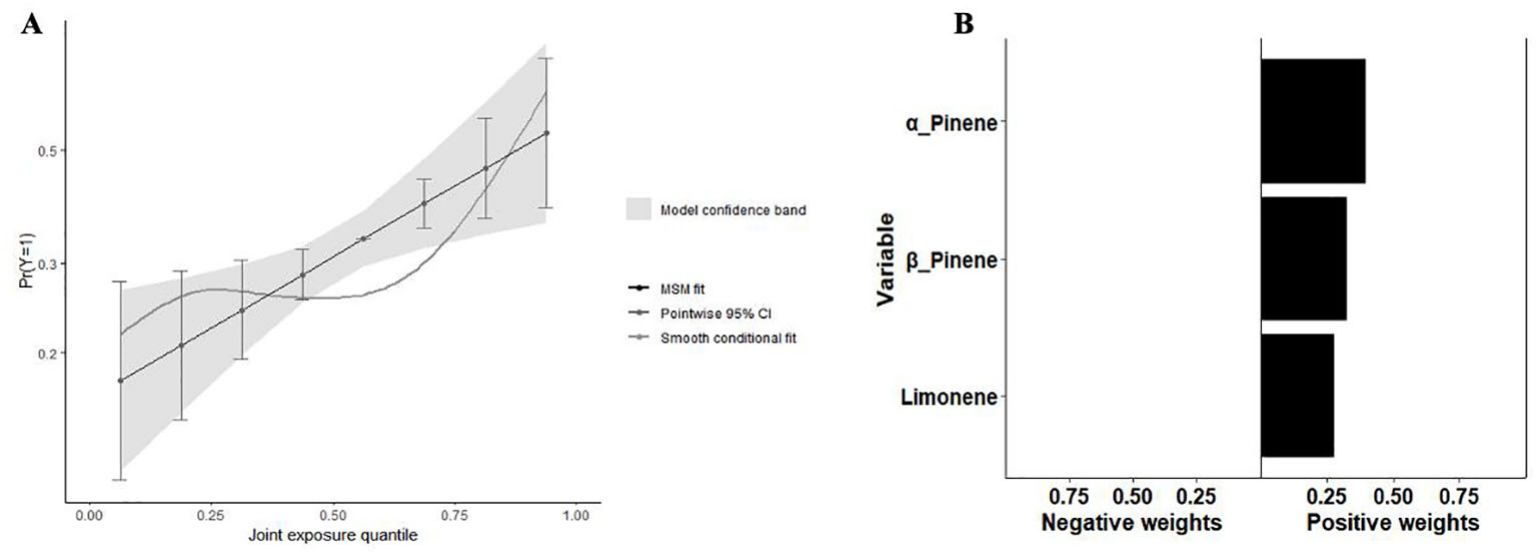
Analysis results of the QGC model. **(A)** The joint effects of terpene on Mets by QGC; **(B)** Weight of each terpene with Mets by QGC.

In **Figure 7A**, the QGC model indicates a significant positive correlation between mixed terpene exposures and Mets (p=0.001). **Figure 7B** shows the relative weights of each monoterpene with Mets and there are significant positive trends between all three terpene derivatives and Mets after adjusting for all covariates. **Supplementary Table S6** shows the overall exposure effect coefficient and confidence interval of the QGC model.

### 3.6 The Mediation of the relationship between terpene compounds and mets by inflammatory effects

**Figure 8:**
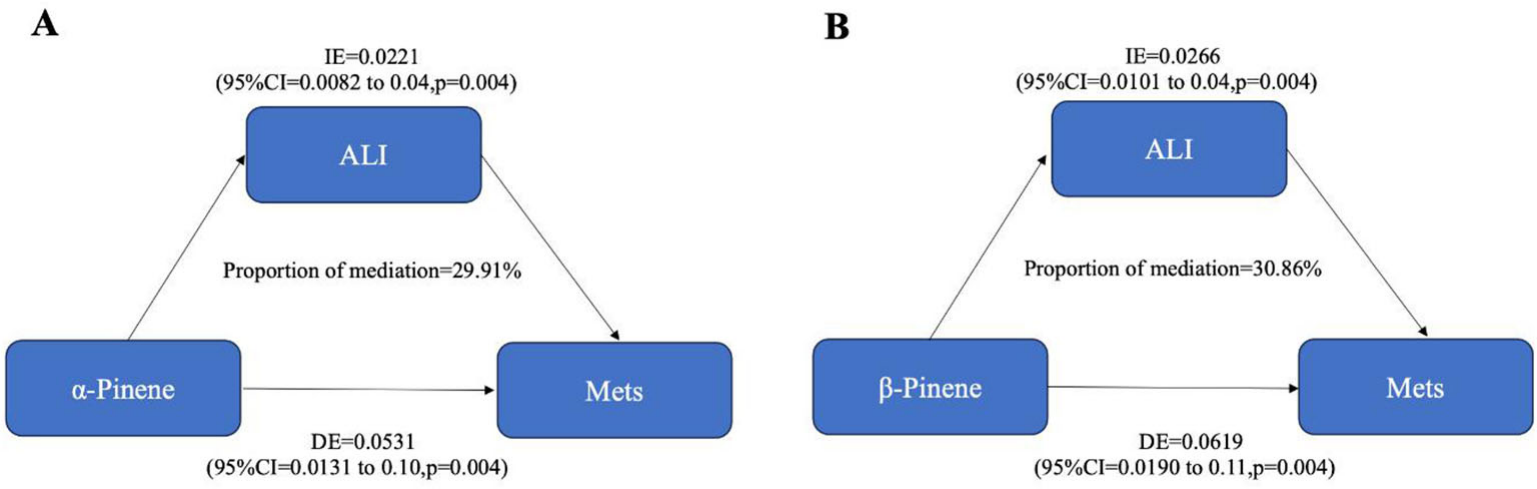
The mediating effects of inflammatory on the relationship between terpene compounds and metabolic syndrome.

We investigated the mediating role of inflammatory effects, as indicated by advanced Cancer Inflammation Index (ALI), on the relationship between limonene, α-pinene, β-pinene, and Mets. As shown in **Figure 8**, our findings indicate that ALI plays a significant mediating role in the relationship between α-pinene and Mets. Specifically, ALI accounts for 29.91% of the overall effect of the positive correlation between α-pinene and Mets. This suggests that the presence of ALI partially explains the relationship between α-pinene and metabolic syndrome. Additionally, our analysis revealed that ALI also serves as a mediator in the relationship between β-pinene and Mets. ALI accounts for 30.86% of the total effect of the positive relationship between β-pinene and Mets. These findings indicate that the influence of β-pinene on metabolic syndrome is, in part, mediated by the presence of ALI (**Table 2**).

**Table 2:** Indirect mediation of inflammatory effects between terpene compounds and the metabolic syndrome (N = 1135).

Pathways	Indirect effect	Mediation proportions and p	
	(β, 95% CI)	95%CIs	
α_Pinene→ ALI→ Mets	**0.026(0.01,0.04)**	**0.30(0.10,0.62)**	0.004**
β_Pinene→ ALI→ Mets	**0.022(0.01,0.04)**	**0.31(0.12,0.40)**	0.004**

The values in bold, 0.026 and 0.022, represent the mediation effect sizes of ALI between α-pinene and β-pinene with Mets, whereas 0.30 and 0.31 indicate the proportions of ALI in the overall positive correlation effects between α-pinene/β-pinene and Mets. **, P < 0.01.

## 4 Discussion

This study used multiple regression, BMKR, and QGC models to conduct a comprehensive analysis and found that exposure to terpenes, alone or in combination, significantly increased the likelihood of developing metabolic syndrome. The stratified analyses revealed significant positive trends among males, individuals under 60 years of age, and those considered to be overweight. Moreover, when subgroup analyses were performed on metabolic syndrome evaluation metrics, mixed terpene exposure demonstrated a positive trend specifically at low HDL levels. Mediation studies have identified a potential mediating role for the Advanced Cancer Inflammation Index in the positive correlation between α-pinene, β-pinene and metabolic syndrome.

Terpene compounds are primarily found in household cleaning products, and research has shown that terpene can be emitted from household cleaning products and persist in the indoor environment for an extended period of time (12). This prolonged exposure to terpenes can pose potential health risks to individuals, as demonstrated by the study of Carolin Rösch (39) et al. Aloha-pinene and limonene are two common terpenes in air fresheners (8). In a laboratory study, concentrations of individual terpenes were observed to range from 10 to 1300 μg/m3 (1.8-234 ppb) over a one-hour period (40). The primary concern regarding terpenes in household cleaning products is their potential to undergo secondary compound reactions when reacting with ozone, leading to the formation of hazardous byproducts such as formaldehyde, secondary organic aerosols (SOA), and ultrafine particles (41, 42). Formaldehyde, acetone, and pinaldehyde are mainly produced in the pinene-ozone reaction, while formaldehyde, 4-acetyl-1-methylcyclohexene, citron, and citral are mainly produced in the reaction between d-limonene and ozone (43). These byproducts can have adverse health effects, such as obesity (44), hypertension (45)cardiovascular diseases (46) and metabolic syndrome (18).

Furthermore, it is well-known that household cleaning products can emit terpenes, which can undergo oxidation with ozone (O3) or hydroxyl (OH) radicals, resulting in the formation of secondary organic aerosols (SOA) (19, 47) and SOA particles formed in the atmosphere from ozone-terpene reactions are an important source of indoor particulate pollution (48–50). Studies have indicated that ambient particulate matter (PM) is composed of SOA and primary particles emitted from tailpipe emissions (51). SOA formed from ozone-terpene reactions is recognized as the predominant contributor to indoor particulate matter (52). Since individuals spend a significant amount of time indoors, exposure to indoor particulate matter may substantially contribute to overall particulate exposure (53). Numerous studies have demonstrated a strong correlation between exposure to particulate matter and various health effects, including elevated blood pressure (54), reduced cardiorespiratory fitness (55, 56), increased blood glucose levels, and an increased risk of diabetes (57). Additionally, a cohort study conducted in China found a positive association between long-term exposure to particulate matter and the risk of Mets (58) and a cohort study conducted in Korea suggested that exposure to organic solvents and SOA may increase the risk of developing metabolic syndrome (59).

What’s more, our analysis revealed a more significant elevation in the risk of developing Mets among males, which could potentially be attributed to the higher exposure to terpenes as flavor additives in cigarette products (60). Sex-stratified analyses revealed that males had a significantly higher prevalence of current smoking (23.9% vs. 19.8% in females, p=0.002) (**Supplementary Table S7**). Furthermore, males exhibited higher serum limonene levels (geometric mean: 1.62 ng/mL vs. 0.45 ng/mL, p=0.000) (**Supplementary Table S3**), potentially linked to terpene-containing vaping products. This aligns with prior findings that males are more likely to use flavored tobacco products, contributing to elevated terpene exposure. We also found a stronger effect of terpene exposure in individuals below the age of 60, possibly due to their higher frequency of exposure to cleaning products in schools and workplaces (61). This could be compounded by impaired melatonin secretion, metabolic imbalances resulting from oxidative stress caused by night shift work and job-related stress (62, 63). Furthermore, we found that terpene exposure had a more pronounced effect in individuals with BMI exceeding 25, indicating a potential exacerbation of the transition from obesity to metabolic syndrome (23).

Finally, we investigated the relationship between terpene exposure and various indicators of metabolic syndrome. The results revealed a highly significant positive correlation between exposure to mixed terpenes and Low HDL levels. We also found positive correlations with elevated triglyceride levels and increased abdominal fat, although the latter correlation did not reach statistical significance (**Figure 6**). It is worth mentioning that a large multicenter retrospective cohort study from China, known as the RCSCD-TCM study, found a strong association between HDL cholesterol ratios and prediabetes, as well as an increased risk of type 2 diabetes (64). These findings were corroborated by a study led by Michel P Herman et al., which demonstrated that elevated triglyceride levels and reduced HDL cholesterol levels are strong risk markers for type 2 diabetes (65). Another cohort study conducted in a southern Italian population comprising 11,987 hypertensive patients revealed an association between low levels of HDL cholesterol and an elevated risk of cardiovascular events (66). This finding aligns with a study conducted by Satoru Kodama et al., which demonstrated that appropriate aerobic exercise could potentially reduce the risk of cardiovascular events through the augmentation of HDL cholesterol levels (67). These findings reinforce our results, providing further evidence for the potential influence of terpene compounds on the risk of metabolic syndrome by precipitating various lipid abnormalities, such as elevated levels of LDL cholesterol and triglycerides, as well as contributing to the occurrence of cardiovascular events.

The Advanced Lung Cancer Inflammation Index (ALI) is an innovative index initially developed as a composite measure of nutrition and inflammation for cancer prognosis, particularly for U.S. community members (68). However, the ALI is now widely utilized as a composite index for assessing nutrition and inflammation in a diverse range of diseases, such as predicting coronary artery disease (29), hypertension (30), and heart failure in the elderly (31). These diseases have close associations with metabolic syndrome (32). Therefore, in this study, we used the ALI to investigate the role of inflammation in the metabolic syndrome by examining the inflammatory effect mediators of terpene compounds, which contribute to the metabolic syndrome.

The study suggests that some of the associations between α-pinene, β-pinene, and the metabolic syndrome are mediated by the ALI. Previous studies have shown that SOA produced by terpene compounds is associated with inflammation *in vivo* (69). A cohort study conducted on older adults in Los Angeles demonstrated a significant and positive association between exposure to secondary organic aerosols (SOA) and interleukin IL-6, a biomarker indicating systemic inflammation (70). Exposure to SOA was also associated with weight loss and aberrant lung inflammation in mice (71). A close association has been established between inflammation, metabolic disruption (24), endothelial stress (72), and insulin resistance (73). Based on these findings, terpene compounds may play a role in promoting inflammatory effects through secondary reactions that generate SOA, thereby increasing the risk of developing metabolic syndrome.

The groundbreaking and innovative outcome of this study is its pioneering revelation of the potential threat terpenes pose to human health. Terpene compounds are found primarily in household cleaning products (prevalent in household cleaning products, air fresheners, and sanitizing products) Surveys of U.S. households have shown that studies indicate that 99.1% of Americans are exposed to terpene-containing scented products on a weekly basis (11). Yet no research has linked terpene compounds ground revealing a threat to physical health and linking it to a disease. Previous studies have shown potential associations between exposure to scented products and adverse health effects, including asthma and asthma exacerbation, but the evidence is weak or insufficient (74), and a UK study has shown that there is a lack of evidence to suggest that current indoor exposures to VOCs, either individually or as a whole, pose a health risk (75). Therefore, the present study fills a gap in the literature by providing the first groundbreaking insight into the effects of terpenes on disease (metabolic syndrome), suggesting that high levels of exposure to terpenes may induce oxidative stress and chronic inflammation, which may adversely affect human health, and provides compelling evidence. Specifically, terpenes have been found to be associated with an increased risk of metabolic syndrome, as indicated by several metabolic markers (such as low levels of high-density lipoprotein and high triglycerides). Terpenoids, which are widely present in cleaning and sanitizing products used in our daily lives, often go unnoticed despite their high frequency of use and extensive application range. This study enhances our understanding of these commonly utilized chemical substances, highlighting their potential hazards. Consequently, further investigation is warranted to establish exposure limits and discern the adverse effects resulting from different levels of exposure.

However, our study also has several limitations that warrant acknowledgment. First, due to the cross-sectional nature of this study, causal inferences cannot be established. Therefore, further case-control or cohort studies are necessary to explore the relationship between terpene exposure and Mets. Second, although we accounted for potential confounding factors as comprehensively as possible, some confounders were unavailable in our database, such as genetic information related to metabolic syndrome, which could not be included in the analysis due to the absence of relevant data in the NHANES database. Third, the exclusion of 85% of NHANES participants with incomplete data may introduce selection bias. The remaining participants exhibited healthier characteristics (e.g., lower BMI and fewer comorbidities), potentially attenuating the observed association between terpene exposure and Mets. To address this limitation, future studies should incorporate advanced methodological approaches, such as imputation techniques, to minimize bias. Fourth, while ALI is a clinically validated composite biomarker for inflammation and nutrition in cancer populations, its applicability in metabolic syndrome remains exploratory. Future research should employ prospective cohort designs to systematically compare ALI with gold-standard inflammatory biomarkers, such as C-reactive protein (CRP) and interleukin-6 (IL-6), in metabolic disorders. Fifth, serum terpene levels were measured at a single time-point, which may fail to capture long-term or cumulative exposure. Terpene concentrations can fluctuate due to seasonal variations in indoor activities (e.g., cleaning frequency) and outdoor ozone levels. Longitudinal studies with repeated measurements are needed to assess chronic exposure and its metabolic effects. Additionally, our analyses did not adjust for multiple testing, which may increase the likelihood of false-positive findings. Future confirmatory studies should apply rigorous correction methods, such as false discovery rate (FDR) control, to enhance robustness. Finally, only three terpene compounds (α-Pinene, β-Pinene, Limonene) were included in this study, as these represent the primary terpenes provided in the NHANES database.

## 5 Conclusions

This study revealed a significant connection between terpene exposure, either alone or in combination, and an increased risk of metabolic syndrome. Specifically, limonene showed the most pronounced effect. Additionally, high exposure to terpene mixtures was linked to low HDL levels. Furthermore, mediation analyses indicate that inflammatory effects could mediate the relationship between terpene exposure and the elevated risk of metabolic syndrome. Therefore, further comprehensive cohort studies are necessary to validate this association.

## Data Availability

The original contributions presented in the study are included in the article/**Supplementary Material**. Further inquiries can be directed to the corresponding author/s.
