# Supplementary material for: Association between exposure to terpene compounds and risk of metabolic syndrome: exploring the potential mediating role of inflammatory response
Source: Front Endocrinol (Lausanne). 2025 May 6;16:1551784. doi: 10.3389/fendo.2025.1551784 (PMC12088976; doi:10.3389/fendo.2025.1551784)
Supplement: Supplementary file 1 [file DataSheet1.docx]

Supplementary Material

**Table S1** Distribution of terpene, NHANES 2013–2014

**Table S2** Posterior inclusion probabilities (PIPs) of BKMR model

**Table S3** Association between terpene and Mets among U.S. adults by sex groups

**Table S4** Association between terpene and Mets among U.S. adults by BMI groups

**Table S5** Association between terpene and Mets among U.S. adults by age groups

**Table S6** Overall exposure effect coefficients and confidence intervals for the QGC model

**Table S7** Demographic and individual characteristics of the study population stratified by sex

**Table S8** Characteristics of Included vs. Excluded Participants

**Figure S1** Spearman correlation coefficients among terpene

**Figure S2** Bivariate exposure-response function plots (Generated using the BKMR software package, which describes the relationship between potential pairwise interactions between a terpene metabolite (at its 25th, 50th, and 75th percentiles) and other metals fixed at its 50th percentile.)

**Figure S3** The single-exposure effect of individual terpenes on the Mets (The estimated values and 95% confidence intervals for all other terpenes were fixed at the 25th, 50th, or 75th percentiles by BKMR)

**Table S1** Distribution of terpene, NHANES 2013–2014

| terpene | Detection frequency | Geometric mean | Mean | Percentile | | | | |
| --- | --- | --- | --- | --- | --- | --- | --- | --- |
|  |  |  |  | 10th | 25th | 50th | 75th | 90th |
| α_Pinene | 72.9 | 0.95 | 0.08 | 0.03 | 0.05 | 0.08 | 0.11 | 0.17 |
| β_Pinene | 73.0 | 0.94 | 0.07 | 0.04 | 0.05 | 0.07 | 0.10 | 0.06 |
| Limonene | 100 | 1.47 | 1.18 | 0.70 | 0.87 | 1.18 | 1.63 | 2.48 |

**Table S2** Posterior inclusion probabilities (PIPs) of BKMR model

| terpene | Total | | |
| --- | --- | --- | --- |
|  | Group | Group PIP | Cond PIP |
| α_Pinene | 1 | 0.875 | 0.4261 |
| β_Pinene | 1 | 0.875 | 0.5738 |
| Limonene | 2 | 0.955 | 1.0000 |

Models were adjusted for age group, sex, race, marital status, BMI index, smoking, drinking, exercise, education level, marital status, ratio of family income to poverty, urine creatinine, hypertension, depression, cardiovascular disease, and hyperuricemia.

**Table S3** Association between terpene and Mets among U.S. adults by sex

| terpene | Female | | male | |
| --- | --- | --- | --- | --- |
|  | Coef(95%CI) | *P* value | Coef(95%CI) | *P* value |
| α_Pinene | 1.44(1.02,2.03) | **0.035*** | 0.92(0.68,1.26) | 0.630 |
| β_Pinene | 0.77(0.53,0.11) | 0.163 | 1.37(0.98,1.92) | 0.060 |
| Limonene | 0.45(1.10,1.89) | **0.007**** | 1.62(1.23,2.13) | **0.000***** |

Models were adjusted for age group, sex, race, marital status, BMI index, smoking, drinking, exercise, education level, marital status, ratio of family income to poverty, urine creatinine, hypertension, depression, cardiovascular disease, and hyperuricemia. * *P* < 0.05; ** *P* < 0.01; *** *P* < 0.001.

**Table S4** Association between terpene and Mets among U.S. adults by BMI

| terpene | BMI<25 | | BMI>=25 | |
| --- | --- | --- | --- | --- |
|  | Coef(95%CI) | *P* value | Coef(95%CI) | *P* value |
| α_Pinene | 2.24(0.72,6.97) | 0.161 | 0.10(0.88,1.39) | 0.366 |
| β_Pinene | 0.28(0.07,1.06) | 0.063 | 1.05(0.83,1.34) | 0.650 |
| Limonene | 1.19(0.54,2.59) | 0.654 | 1.44(1.19,1.73) | **0.000***** |

Models were adjusted for age group, sex, race, marital status, BMI index, smoking, drinking, exercise, education level, marital status, ratio of family income to poverty, urine creatinine, hypertension, depression, cardiovascular disease, and hyperuricemia. * *P* < 0.05; ** *P* < 0.01; *** *P* < 0.001.

**Table S5** Association between terpene and Mets among U.S. adults by age

| terpene | Age>=60 | | Age<60 | |
| --- | --- | --- | --- | --- |
|  | Coef(95%CI) | *P* value | Coef(95%CI) | *P* value |
| α_Pinene | 1.05(0.72,1.54) | 0.776 | 1.08(0.82,1.442) | 0.577 |
| β_Pinene | 0.92(0.63,1.35) | 0.679 | 1.16(0.86,1.56) | 0.316 |
| Limonene | 1.35(0.99,1.83) | 0.053 | 1.62(1.28,2.06) | **0.000***** |

Models were adjusted for age group, sex, race, marital status, BMI index, smoking, drinking, exercise, education level, marital status, ratio of family income to poverty, urine creatinine, hypertension, depression, cardiovascular disease, and hyperuricemia. * *P* < 0.05; ** *P* < 0.01; *** *P* < 0.001.

**Table S6** Overall exposure effect coefficients and confidence intervals for the QGC model

|  | Estimate Std. Error Lower CI | | Upper CI Z value Pr(>lzl) | |
| --- | --- | --- | --- | --- |
| (Intercept) | 1.274 | 0.128 -1.525 | -1.023 | -9.955  **0.000***** |
| psi1 | 0.231 | 0.654 0.1004 | -0362 | 3.462  **0.000***** |

**Table S7** Demographic and individual characteristics of the study population stratified by sex (N=1,135)

|  | Male (N=594) | Female (N=541) | P-value |
| --- | --- | --- | --- |
| Age group (n(%)) |  |  |  |
| 20~60years | 412 (69.4%) | 382 (70.6%) | 0.694 |
| Over 60 years | 182 (30.6%) | 159 (29.4%) |  |
| Race (n(%)) |  |  |  |
| Mexican American | 65 (10.9%) | 68 (12.6%) | 0.489 |
| Other Hispanic | 46 (7.7%) | 48 (8.9%) |  |
| Non-Hispanic White | 303 (51.0%) | 282 (52.1%) |  |
| Non-Hispanic Black | 107 (18.0%) | 92 (17.0%) |  |
| Other Race - Including Multi-Racial | 73 (12.3%) | 51 (9.4%) |  |
| Marital status (n(%)) |  |  |  |
| Married/Living with partner | 395 (66.5%) | 279 (51.6%) | <0.001*** |
| Never married | 119 (20.0%) | 109 (20.1%) |  |
| Widowed/Divorced/Separated | 80 (13.5%) | 153 (28.3%) |  |
| Education level (n(%)) |  |  |  |
| Under high school | 116 (19.5%) | 76 (14.0%) | 0.02 |
| Hight school or equivalent | 144 (24.2%) | 122 (22.6%) |  |
| Above high school | 334 (56.2%) | 343 (63.4%) |  |
| Ratio of Family Income to Poverty (n(%)) |  |  |  |
| Poverty | 118 (19.9%) | 112 (20.7%) | 0.782 |
| Above Poverty | 476 (80.1%) | 429 (79.3%) |  |
| Smoking (n(%)) |  |  |  |
| Never | 263 (44.3%) | 310 (57.3%) | <0.001*** |
| Ever | 189 (31.8%) | 123 (22.7%) |  |
| Current | 142 (23.9%) | 108 (20.0%) |  |
| Drinking (n(%)) |  |  |  |
| Less than 12 alcoholic beverages/1 year | 453 (76.3%) | 490 (90.6%) | <0.001*** |
| 12 alcoholic beverages/1 year or more | 141 (23.7%) | 51 (9.4%) |  |
| Exercise (n(%)) |  |  |  |
| Not exercising regularly | 293 (49.3%) | 261 (48.2%) | 0.76 |
| Exercise regularly | 301 (50.7%) | 280 (51.8%) |  |
| Hypertension (n(%)) |  |  |  |
| Yes | 211 (35.5%) | 205 (37.9%) | 0.444 |
| No | 383 (64.5%) | 336 (62.1%) |  |
| Depression (n(%)) |  |  |  |
| Yes | 36 (6.1%) | 66 (12.2%) | <0.001*** |
| No | 558 (93.9%) | 475 (87.8%) |  |
| Cardiovascular Disease (n(%)) |  |  |  |
| Yes | 74 (12.5%) | 38 (7.0%) | 0.003** |
| No | 520 (87.5%) | 503 (93.0%) |  |
| Hyperuricemia (n(%)) |  |  |  |
| Yes | 125 (21.0%) | 102 (18.9%) | 0.397 |
| No | 469 (79.0%) | 439 (81.1%) |  |
| Nonalcoholic Fatty Liver Disease (n(%)) |  |  |  |
| Yes | 305 (51.3%) | 338 (62.5%) | <0.001*** |
| No | 289 (48.7%) | 203 (37.5%) |  |
| BMI index (n(%)) |  |  |  |
| Mean (SD) | 3.33 (0.200) | 3.37 (0.252) | 0.001** |
| Median [Min, Max] | 3.31 [2.73, 4.31] | 3.35 [2.83, 4.35] |  |
| Urine creatinine (n(%)) |  |  |  |
| Mean (SD) | 4.65 (0.693) | 4.32 (0.770) | <0.001*** |
| Median [Min, Max] | 4.76 [2.08, 6.26] | 4.38 [1.61, 6.30] |  |
| α_Pinene (ng/mL) |  |  |  |
| Tertile1[n(%)] | 139 (23.4%) | 145 (26.8%) | 0.345 |
| Tertile2[n(%)] | 152 (25.6%) | 140 (25.9%) |  |
| Tertile3[n(%)] | 161 (27.1%) | 124 (22.9%) |  |
| Tertile4[n(%)] | 142 (23.9%) | 132 (24.4%) |  |
| β_Pinene (ng/mL) |  |  |  |
| Tertile1[n(%)] | 145 (24.4%) | 149 (27.5%) | 0.652 |
| Tertile2[n(%)] | 155 (26.1%) | 130 (24.0%) |  |
| Tertile3[n(%)] | 150 (25.3%) | 135 (25.0%) |  |
| Tertile4[n(%)] | 144 (24.2%) | 127 (23.5%) |  |
| Limonene (ng/mL) |  |  |  |
| Tertile1[n(%)] | 130 (21.9%) | 156 (28.8%) | 0.016** |
| Tertile2[n(%)] | 151 (25.4%) | 136 (25.1%) |  |
| Tertile3[n(%)] | 148 (24.9%) | 134 (24.8%) |  |
| Tertile4[n(%)] | 165 (27.8%) | 115 (21.3%) |  |

Note: *, P < 0.05; **, P < 0.01; ***, P < 0.001. P-t, p-value for trend.

**Table S8**  Characteristics of Included vs. Excluded Participants

| Characteristic | Included (n=1,135) Excluded (n=9,662) p-value | |
| --- | --- | --- |
| Age (years), mean±SD | 48.2±16.1 | 49.8±17.3 0.004* |
| Male, % | 51.3 | 49.1 0.102 |
| BMI (kg/m²), mean±SD | 27.8±5.9 | 29.1±7.2 <0.001*** |
| College graduate, % | 45.2 | 31.8 <0.001*** |
| Hypertension, % | 28.1 | 34.9 <0.001*** |
| Current smoker, % | 18.7 | 22.4 0.003** |

Note: *, P < 0.05; **, P < 0.01; ***, P < 0.001. P-t, p-value for trend.


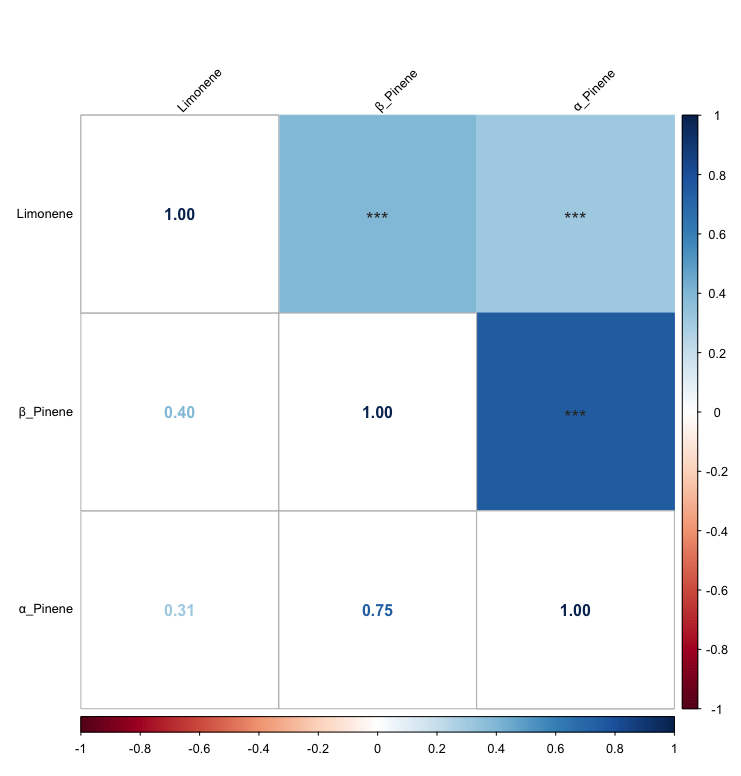


**Figure S1** Spearman correlation coefficients among terpenes


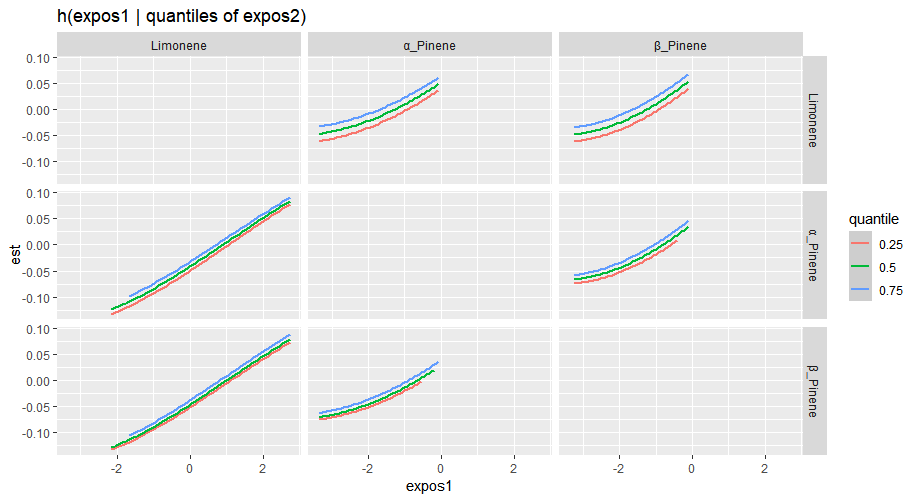


**Figure S2** Bivariate exposure-response function plots (Generated using the BKMR software package, which describes the relationship between potential pairwise interactions between a terpene metabolite (at its 25th, 50th, and 75th percentiles) and other metals fixed at its 50th percentile.)


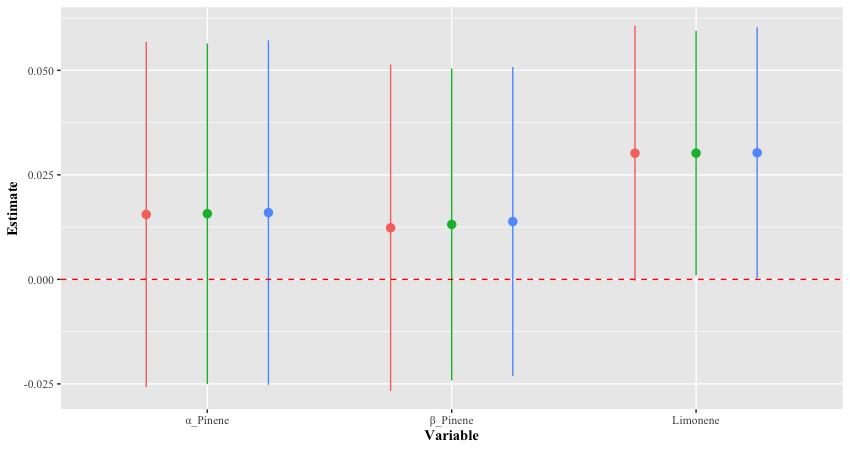


**Figure S3** The single-exposure effect of individual terpenes on the Mets (The estimated values and 95% confidence intervals for all other terpenes were fixed at the 25th, 50th, or 75th percentiles by BKMR)
